# The unique and compensatory effects of home and classroom learning activities on Migrant and Seasonal Head Start children’s Spanish and English emergent literacy skills

**DOI:** 10.3389/fpsyg.2022.1016492

**Published:** 2022-11-25

**Authors:** Rufan Luo, Lulu Song

**Affiliations:** ^1^School of Social and Behavioral Sciences, New College of Interdisciplinary Arts and Sciences, Arizona State University, Phoenix, AZ, United States; ^2^Department of Psychology, Rutgers University-Camden, Camden, NJ, United States; ^3^Department of Early Childhood Education/Art Education, Brooklyn College, The City University of New York, New York, NY, United States

**Keywords:** dual language learners, Migrant and Seasonal Head Start, emergent literacy, home language and literacy activities, classroom language and literacy activities

## Abstract

Children of migrant and seasonal farmworkers (MSFW) are among the most underprivileged, underserved groups in the United States. The current study examined how home and classroom language and literacy experiences uniquely and interactively contributed to MSFW children’s emergent literacy skills in English and Spanish. Participants were 255 Spanish-English dual language learning children (*M*_age_ = 49 mon; 98.3% Latino/Hispanic) and their parents and 47 teachers, drawn from the Migrant and Seasonal Head Start (MSHS) Study. Parents reported how often the target children engaged in language and literacy activities (i.e., teaching letters, words, or numbers, book-reading, singing, and storytelling) with their family members. Teachers reported how often the target children engaged in classroom language and literacy activities (e.g., book-reading, learning letters, retelling stories, etc.). Children’s emergent literacy skills in English and Spanish were assessed by standard tests. After controlling for demographic variables, home and classroom language and literacy activities uniquely predicted children’s emergent literacy skills in Spanish, but not in English. Additionally, home and classroom activities compensated one another in supporting children’s English and Spanish emergent literacy development. That is, language and literacy activities in one context showed a stronger effect for children who experienced less frequent activities in the other context. Together, these findings shed light on ways to support MSFW children’s emergent literacy skills and reveal the importance of integrating and connecting home and school learning experiences.

## Introduction

In the United States, there are 2.5–3 million migrant and seasonal farmworkers (MSFW; National Center for Farmworker Health (NCFH), 2020), most of whom are foreign born (75%), self-identified as Hispanic/Latino (83%), and used Spanish as their primary language (77%; National Center for Farmworker Health (NCFH), 2020). They have an average education level of 8th grade, and approximately one third of them are living below the poverty line (National Center for Farmworker Health (NCFH), 2020). Young children of MSFW families are among the most underprivileged, underserved groups in the United States (Mathur and Parameswaran, 2012), facing developmental obstacles such as food insecurity, unstable and crowded housing, language and cultural barriers, and limited access to educational and healthcare services (Perreira et al., 2006; Barrueco, 2012; Tavassolie et al., 2018). To date, most studies have focused on MSFW children’s mental and physical health (Kupersmidt and Martin, 1997; Beltran, 2010; Taylor and Ruiz, 2017), with a scarcity of work on their development of early language and literacy skills. As a branch of the Head Start program (a federal program providing free early childhood education to low-income families), the Migrant and Seasonal Head Start (MSHS) program has been designed to offer high-quality, culturally appropriate child development and family support to MSFW families across 38 states in the United States (Early Childhood Learning and Knowledge Center (ECLKC), 2022), providing a valuable context for studying MSFW children’s language and literacy development and experiences.

Children’s emergent literacy skills, such as their knowledge of letters and words, phonological awareness, and print concepts, are important precursors of future reading skills and academic success (Lonigan et al., 2000). Most MSFW children are Spanish-English dual language learners (DLLs), who are exposed to Spanish at home and English at preschool during the months they are enrolled in MSHS programs (Mathur and Parameswaran, 2012). Given their limited English experiences, many MSFW children struggle with emergent literacy in English, which could later become a barrier for school achievement (Tavassolie et al., 2018). A study of MSFW children in Florida showed that, even though children made progress in their English over time, only 43% of the children reached the developmental benchmark in English at the end of preschool; and in kindergarten, 52% and 23% of MSFW children were at high and medium risk in their development of English emergent literacy skills (Tavassolie et al., 2018). Additionally, MSFW parents express concerns regarding their children’s Spanish loss (Smith and Johnson, 2019). Spanish is critical for children to develop their Latino identity, learn about their culture and heritage, and communicate with family members. Yet, MSFW children tend to use less Spanish after being exposed to English in preschool (Smith and Johnson, 2019).

Language and literacy activities in both the home and classroom contexts offer children important opportunities to develop emergent literacy skills (Hammer et al., 2014; Piasta, 2016). However, very few studies have examined MSFW children’s home and classroom experiences simultaneously. The current study asked how home and classroom language and literacy activities uniquely predicted MSFW children’s emergent literacy skills in English and Spanish; and whether home and classroom activities interacted with each other in their contributions to children’s emergent literacy skills.

### Home language and literacy activities and children’s emergent literacy skills

Ample research has demonstrated the effect of home language and literacy experiences on children’s emergent literacy skills (e.g., Reese et al., 2010; Farver et al., 2013). In particular, the frequency of language and literacy activities (e.g., book-reading, teaching letters and words, storytelling, and singing songs) is found to relate to children’s emergent literacy skills (Reese et al., 2000; Raikes et al., 2006; Rodriguez and Tamis-LeMonda, 2011).

Book-reading exposes children to language input that is diverse, complex, and cognitively demanding (Peterson and McCabe, 1994; Soderstrom and Wittebolle, 2013; Tamis-LeMonda et al., 2018). Both correlational and intervention work has revealed the benefits of frequent book-reading for early language and literacy development (Bus et al., 1995; Raikes et al., 2006; Noble et al., 2019). Studies with low-income, Latino families also documented the links between book-reading frequency and children’s emergent literacy skills in both English and Spanish (Farver et al., 2013; Gonzalez et al., 2017; Shen and Del Tufo, 2022). For instance, in a sample of Latino Head Start children, parents’ engagement with children in literacy activities in English and Spanish predicted children’s emergent literacy skills in both languages (Farver et al., 2013). Importantly, parent–child book-reading in one language might benefit children’s emergent literacy skills in the other language. One study showed that the frequency of mother–child book-reading, which was primarily conducted in Spanish only or both English and Spanish, predicted Latino children’s receptive vocabulary in English during preschool years (Gonzalez et al., 2017).

Other than book-reading, parental engagement in code-related activities such as teaching children how to read and write letters and words also support the development of emergent literacy skills (Sénéchal and Lefevre, 2002, 2014; Haney and Hill, 2004; Hood et al., 2008; Inoue et al., 2018). For example, a three-year longitudinal study has suggested that the frequency of parental teaching of literacy skills predicted children’s concurrent emergent literacy skills at preschool and their reading and spelling skills in 1^st^ and 2^nd^ grades (Hood et al., 2008). Similar findings have been observed in monolingual Spanish-speaking samples. For example, a study with low-socioeconomic status Chilean families found that mothers who frequently taught children how to read and write letters had children who showed better letter-word identification skills (Mendive et al., 2020).

Additionally, learning activities that do not rely on print materials, such as storytelling and singing, are highly valued in Latino and/or MSFW families as essential ways to support children’s language and literacy development and convey cultural lessons (Luo and Tamis-LeMonda, 2019; van der Pluijim et al., 2019). It is therefore critical to include these non-print activities in the examination of home experiences. Indeed, studies using combined measures of children’s engagement in book-reading, storytelling, and singing have found positive associations between the frequency of these activities and children’s early language and/or emergent literacy skills in infancy and preschool years (Rodriguez et al., 2009; Tamis-LeMonda et al., 2019; Song et al., 2022).

Fewer studies have examined children’s language and literacy experiences in MSFW families. Constraints such as long working hours, high illiteracy rates, limited access to learning materials, and unfamiliarity with the U.S. education system present challenges for MSFW parents to support their children’s early literacy development (Mehta et al., 2000; Perreira et al., 2006; Tavassolie et al., 2018). Nonetheless, MSFW parents value education and have high dedication to and expectation for their children (O’Brien et al., 2011; Barrueco, 2012; Smith and Johnson, 2019). Qualitative research has shown that MSFW parents engaged their children in a variety of reading and writing activities, including but not limited to reading books, messages, and letters from their families, reading and writing alphabetic letters and children’s names, reading the Bible, and telling stories (Lynch, 2008; Purcell-Gates, 2013). In a study of 48 MSHS children, researchers examined children’s emergent literacy skills and their home literacy experiences (e.g., book-reading frequency, access to books in English and Spanish), and found that the composite score of home literacy experiences predicted children’s emergent literacy skills in their dominant language (Ezell et al., 2000). Intervention studies aiming to promote language and literacy activities in MSFW or migrant families have also shown positive effects on children’s early language and literacy skills (Boyce et al., 2010; St. Clair et al., 2012).

### Classroom language and literacy activities and children’s emergent literacy skills

The quantity/frequency of classroom language and literacy activities has been found to support children’s emergent literacy skills (Xue and Meisels, 2004; Connor et al., 2006; Guarino et al., 2006; Zucker et al., 2013). For example, in a study with a culturally and linguistically diverse sample, pre-k children who spent more time in teacher-directed activities such as book-reading showed greater gains in their emergent literacy skills over the school year (Pianta et al., 2020). Longitudinal studies have also found that the duration or frequency of language and literacy activities in preschool or kindergarten predicted children’s language and emergent literacy growth from pre-k to kindergarten (Christopher and Farran, 2020) and their gains in reading skills from kindergarten to 5^th^ grade (Sonnenschein et al., 2010). These findings highlight the facilitative role of classroom language and literacy activities in children’s early literacy development.

Most children in the MSHS programs are DLLs and speak Spanish as their primary language (Stechuk and Burns, 2005). Yet, research on Spanish-English DLLs’ classroom language and literacy experiences is still limited. Like their monolingual peers, DLLs benefit from frequent, high-quality language and literacy instructions (Gersten and Geva, 2003; Graves et al., 2004; Gersten et al., 2005; Baker et al., 2006; Cirino et al., 2007). At the same time, it is crucial for teachers to provide DLLs with culturally and linguistically responsive instructions and support their home language development (Castro et al., 2011; Sawyer et al., 2016). For example, Head Start teachers’ instructional support in the DLLs’ home language (e.g., quantity of Spanish use, instructional strategies such as questioning and literacy materials in Spanish) has been found to predict DLLs’ home language skills (White et al., 2020). Other studies comparing bilingual and English-only programs have also suggested that bilingual programs support children’s Spanish development without slowing down their English acquisition (Collier and Thomas, 2004; Rolstad et al., 2005; Barnett et al., 2007; Figueras-Daniel and Li, 2021). Nonetheless, observations of preschool teachers’ classroom practices with DLLs have suggested that teachers tend to use few linguistically responsive practices (e.g., providing key words in children’s home language, giving children opportunities to use both English and the home languages) and more basic, low-quality language and literacy instructions (e.g., not using many open-ended questions or advanced vocabularies) with DLLs (Justice et al., 2008; Sawyer et al., 2016).

To date, few studies have examined teachers’ language and literacy practices in MSHS classrooms in relation to children’s developmental outcomes. One intervention study found that training teachers to use high-quality instructions (e.g., building children’s vocabulary, engaging children in book-reading, and implementing classroom activities in a playful and effective way) during classroom language and literacy activities promoted MSHS children’s emergent literacy growth in English and Spanish (Solari et al., 2016).

### The unique and interactive effects of home and classroom experiences

Most research has examined the effects of children’s home and classroom language and literacy experiences separately, without asking how these two components of children’s learning experiences uniquely contribute to the development of emergent literacy skills and whether they interact with one another. The bioecological model posits that children’s immediate contexts (e.g., home and classroom settings) are not independent. Rather, various developmental contexts interact with each other in their contribution to child outcomes (Bronfenbrenner and Morris, 2006). Similarly, the multisystemic approach proposes that child development unfolds within an interconnected system of individual, family, and extra-familial (e.g., school, community) factors (Barrueco, 2012). It is therefore necessary to consider children’s home and school experiences simultaneously in minority, marginalized populations.

Studies examining the unique effect of home and classroom language and literacy experiences have yielded mixed findings. One study examined the effect of home and classroom literacy experiences (e.g., book-reading frequency and the availability of literacy materials in the home and classroom settings) on the emergent literacy skills of children enrolled in MSHS programs and found that home literacy experiences was a stronger predictor than classroom literacy experiences (Ezell et al., 2000). However, a study with Turkish 5-year-olds found that classroom literacy experiences (e.g., the availability of books, book-reading and early writing activities, etc.), but not home literacy experiences (e.g., number of books, book-reading frequency, etc.), predicted children’s emergent literacy skills 4 months later (Altun et al., 2018). Other studies have documented the unique roles of both home and classroom experiences. For example, a reading intervention study compared three conditions, a classroom only condition in which teachers were trained to read to children using the dialogic reading approach, a classroom plus home condition in which both teachers and parents were trained to read to children using the same approach, and a control condition (Whitehurst et al., 1994). While both treatment conditions improved children’s early language skills, the classroom plus home condition had a stronger effect than the classroom only condition. Another study found that 3-year-old children who had better home literacy experiences and whose preschool center had higher levels of average child ability (a potential indicator of center quality) showed more advanced literacy skills at 1^st^ and 3^rd^ grades (Melhuish et al., 2008).

To date, no studies to our knowledge have examined the interaction between home and school language and literacy activities in relation to MSFW children’s emergent literacy skills. However, studies with other populations have suggested that home and classroom experiences may shape child development in a compensatory manner, such that rich, high-quality language and literacy experiences in one context may compensate the poor experiences in the other context (Magnuson et al., 2004; McCartney et al., 2007; Crosnoe et al., 2010; Vernon-Feagans et al., 2013). For example, Vernon-Feagans et al. (2013) found children who received less complex maternal language input at home to benefit more from positive caregiver–child verbal interactions. Other studies have also shown that high-quality classroom experiences matter more for children with poorer home learning experiences due to factors such as low-income, low maternal education, and single parenthood (Magnuson et al., 2004; McCartney et al., 2007; Crosnoe et al., 2010). Home literacy experiences can also compensate the lack of language and literacy support in the classroom context. For example, one study found that DLL children’s engagement in home literacy activities in their heritage language predicted their vocabulary growth in the societal language, but only for those who received low levels of classroom language stimulation (Willard et al., 2021).

In contrast to the compensatory hypothesis, some studies found little evidence that children from more disadvantaged families would benefit more from high-quality classroom experiences (Burchinal et al., 2000). There is even some evidence suggesting that children with stimulating home experiences might be better prepared for learning in the classroom, indicating a complementary relationship of home and school experiences. A study with a low-income sample found that high-quality childcare positively predicted children’s emergent literacy skills only for those children exposed to high cognitive stimulation at home (Votruba-Drzal et al., 2004). Similarly, a study of Head Start children suggested a greater effect of classroom quality on children’s problem solving and reasoning for those children with better home learning experiences (e.g., frequent language and literacy activities, abundant learning materials and toys, and warm, non-punitive parenting behaviors; Bryant et al., 1994). Perhaps, children need to reach a certain skill level before taking advantage of their classroom experiences (Vygotsky, 1978), and linguistically and cognitively stimulating home experiences play a key role in helping children achieve the threshold.

### The current study

To better understand the unique and interactive effects of home and classroom literacy activities on MSHS children’s emergent literacy skills, we asked two research questions:

1. How do home and classroom language and literacy activities uniquely contribute to MSHS children’s emergent literacy skills in English and Spanish? We hypothesized that frequencies of language and literacy activities in both the home and classroom settings would account for unique variances in MSHS children’s emergent literacy skills.
2. Do home and classroom language and literacy activities interact with each other in their contributions to children’s English and Spanish literacy skills? If home and classroom activities benefit child development in a compensatory way, we would expect classroom language and literacy activities to show a stronger positive effect for children who experienced less frequent language and literacy activities at home, and vice versa. Alternatively, if home and classroom activities work in a complementary way, we would expect the effect of classroom activities to be stronger for those children who more frequently engaged in language and literacy activities at home.

## Methods

### Participants

Participants were drawn from the Migrant and Seasonal Head Start (MSHS) Study (Caswell et al., 2020), which aimed to understand the characteristics of MSHS programs, children, and families, the quality of MSHS services and practices, and the relation between MSHS characteristics and the outcomes of children and families (Caswell et al., 2019). The study involved a nationally representative sample of 122 MSHS classrooms, 234 lead and assistant teachers, 873 children, and 778 parents (Caswell et al., 2019). Data was collected between January 2017 and January 2018, *via* MSHS staff surveys, parent interviews, classroom observations, and direct child assessments.

Of the original 873 children, 255 had valid data on emergent literacy skills in English and/or Spanish, parental report of home language and literacy activities, and teachers’ report of classroom language and literacy activities. Given that children were only assessed in English and Spanish, we further excluded 20 preschoolers exposed to a home language other than English or Spanish. Thus, the final analytic sample included 235 children and their parents (one parent of each child) and 47 lead teachers. Table 1 presents the demographic information of the sample. On average, children (51.49% males) were 49 months of age (*SD* = 9.17) at the time of assessment. Almost all of them (98.3%) were identified by their parents as Latino/Hispanic. Most participating parents were mothers (88.9%) and had elementary to high school education levels (62.4%). About 82% of the parents reported using all Spanish or more Spanish than English with their children. The teachers’ education levels ranged from high school to graduate education (see Table 1 for more details).

**Table 1:** Descriptive statistics for demographic variables, home and classroom language and literacy activities, and children’s emergent literacy skills in English and Spanish.

			*Mean or Percentage*	*SD*	*Range^a^*	Missing (%)
**Child characteristics** (*n* = 235)						
	Gender (male)		51.49%			0%
	Latino/Hispanic		98.29%			0.43%
	Child age in month at English assessment		49.10	9.17		1.30%
	Child age in month at Spanish assessment		49.08	9.18		0.90%
**Family characteristics** (*n* = 235)						
	Total number of children living in the household		2.01	1.30		
	Parent relationship with the child					
		Mother	88.94%			
		Father	9.79%			
		Grandparents	0.43%			
		Other	0.85%			
	Parental education^b^				1–21.5	0.43%
		No school	1.71%			
		1th–6th grade	22.7%			
		7th–12th grade, no diploma	40.6%			
		High school diploma/Equivalent	21.8%			
		Vocational/technical school	2.1%			
		Some college, no degree	8.6%			
		Bachelor’s degree or some graduate school without a degree	2.6%			
	Parental language use with the target child					7.66%
		All English	0.46%			
		More English than Spanish	14.29%			
		Same amount of Spanish and English	3.23%			
		More Spanish than English	32.72%			
		All Spanish	49.31%			
**Teacher characteristics** (*n* = 47)						
	Teacher educational level					*0%*
		High school diploma	17.02%			
		Vocational/technical school	12.77%			
		Associate’s degree	38.30%			
		Bachelor’s degree	25.53%			
		Higher than Bachelor’s degree	6.38%			
	Teacher instructional language (1-English Completely, 5-Spanish completely)		2.57	1.25	1–5	2.13%
**Key variables**						
	Frequency of home language and literacy activities (1-zero days, 4-5 to 7 days a week)		2.87	0.71	1–4	0%
		Teach letters, words, or numbers	2.93	0.80	1–4	
		Read or look at books	2.85	0.99	1–4	
		Tell stories	2.71	1.06	1–4	
		Sing songs	3.00	1.05	1–4	
		Frequency of classroom language and literacy activities (1-never, 6-every day)	5.21	0.86	2.5–6	0%
		Learn the names of letters	5.52	1.05	1–6	
		Practice writing the letters of the alphabet	4.57	1.60	1–6	
		Discuss new words	5.49	0.86	3–6	
		Work on phonics (e.g., rhyming, sounds of letters)	5.13	1.39	1–6	
		Listen to you read stories	5.85	0.47	4–6	
		Retell stories	5.23	1.20	1–6	
		Learn about conventions of print (e.g., left to right orientation)	5.23	1.46	1–6	
		Write children’s own name	4.62	1.73	1–6	
	Child English emergent literacy skills (standard scores)		83.69	12.84	32–134	1.30%
	Child Spanish emergent literacy skills (standard scores)		94.79	12.42	45–137	0.90%

^a^Minimum and maximum values of identifiable information (i.e., age, household size) are not released to protect data confidentiality.

^b^According to the MSHS dataset, parental education was coded as: 1-no school, 2-preschool, 3-kindergarten, 4 to 14-1st grade to 11th grade, 15–12th grade without a diploma, 16-high school diploma or equivalent, 17-vocational/technical program, 18-vocational/technical diploma, 19-some college, no degree, 20-Associate’s degree, 21.5- Bachelor’s degree or some graduate school without a degree, 23-Master’s degree, 24-Doctoral degree, and 25-professional degree.

### Measures

#### Home language and literacy activities

Parents were interviewed about how many days in the past week they themselves or someone in the family engaged in each of the four types of activities in any language with the target child: teaching the child letters, words, or numbers, reading or looking at books, singing songs, and telling stories (1– zero days, 2 – 1 to 2 days a week, 3 – 3 to 4 days a week, and 4 – 5 to 7 days a week). An average score across these 4 activities was calculated, with higher scores indicating more frequent language and literacy activities at home (*M* = 2.87, *SD* = 0.71, *Range* = 1–4, Cronbach’s Alpha = 0.69).

#### Classroom language and literacy activities

Teachers were asked about how often children in their class engaged in each of the eight types of language and literacy activities in any language in a survey: learning the names of letters, writing children’s own names, learning about the conventions of print, retelling stories, listening to stories read by teachers, working on phonics, discussing new words, and practicing writing alphabets (1 – never, 2 – about once a month or less, 3 – two to three times a month, 4 – once or twice a week, 5 – three to four times a week, 6 – every day). An average score was calculated to indicate how frequently children engaged in these eight activities (*M* = 5.46, *SD* = 0.57, *Range* = 2.5–6, Cronbach’s Alpha = 0.80).

#### Emergent literacy skills

Children’s emergent literacy skills in English and Spanish were assessed using the Letter-Word Identification (English) and Identificación de letras y palabras (Spanish) scales on the Woodcock-Muñoz Language Survey–Revised Normative Update (WMLS-R NU; Woodcock et al., 2005). These assessments examine children’s knowledge of the alphabet and their ability to read single words (Cronbach’s Alpha = 0.87 for English and 0.85 for Spanish; Caswell et al., 2019). All children were assessed in both English and Spanish, starting with the child’s dominant language as reported by their parents. The English and Spanish standard scores were used for analyses. It is important to note that the norms were based on English-speaking and Spanish-speaking monolingual samples. Although the standard scores captured individual differences among children, they likely underestimated DLLs’ skill levels and must be interpreted with caution.

#### Covariates

A group of demographic variables were controlled for in the analyses. At the child/family level, we controlled for children’s age, gender, the total number of children living in the household, parental education, and parents’ relative language use with the child (1-All English, 3-Same amount of Spanish and English, and 5-All Spanish). According to the MSHS dataset, parental education was coded based on a scale, ranging from 1 (no school) to 25 (professional degree; see more details in Table 1 Notes). At the classroom level, we included teachers’ education level (1-less than high school diploma, 5-higher than Bachelor’s degree) and instructional language. Teachers reported on the language(s) they used when teaching children, reading to children, and presenting information, on a scale from 1 (English completely) to 5 (Spanish completely). An average score was calculated to indicate teachers’ relative use of English and Spanish (*M* = 2.57, *SD* = 1.25, *Range* = 1–5). Across the sample, teachers used slightly more English than Spanish.

## Analytic plan

As shown in Table 1, the proportions of missing values were low, ranging from 0 to 7.7%. The Little’s Missing Completely at Random (MCAR) test suggested that data were missing completely at random (*Chi^2^* = 18.63, *p* = 0.91). Multiple imputation was conducted in STATA 17.0 to handle missing values among the control variables (Stata Corp, 2021). The multiple imputation model included all covariates, with and without missing values, such that the missing values were predicted based on existing data. Twenty imputed datasets were generated, and findings were based on pooled estimates.

Because children and families were nested within classrooms, multilevel modeling was conducted using the *mixed* command of STATA 17.0 to examine the effects of child-level (i.e., home language and literacy activities) and classroom-level (i.e., classroom language and literacy activities) factors, accounting for variation between classrooms. First, unconditional models were conducted to estimate the amount of variance explained by differences among individual children (Level 1) and classrooms (Level 2). Second, to examine the contributions of home and classroom language and literacy activities to children’s emergent literacy skills (Research Question 1), a set of two-level random intercept models were estimated, using home language and literacy activities as the key predictor at Level 1, and classroom language and literacy activities as the key predictor at Level 2. Models were estimated separately for English and Spanish literacy skills. Finally, to examine whether home and classroom activities interact with each other in their contributions to emergent literacy skills (Research Question 2), a cross-level interaction term between the home and classroom language and literacy activities was added to the models described above. When the interaction term was significant, we further estimated the conditional effects of classroom language and literacy activities, when the frequency of home language and literacy activities was set to be either low (i.e., 15^th^ percentile of the analytic sample) or high (i.e., 85^th^ percentile). The *nlcom* command of STATA was used after model estimation to compute point estimates, standard errors, *p* values, and confidence intervals for different combinations of parameter estimates (e.g., the effect of one predictor given a specific value of another predictor; Stata Corp, 2021). Likewise, we also estimated the effects of home language and literacy activities, when the frequency of classroom activities was low or high (i.e., 15^th^ and 85^th^ percentiles). In each model, covariates of the home and classroom contexts were included at Level 1 (i.e., child age, gender, parental education, parental Spanish use, total number of children in the household) and Level 2 (i.e., teachers’ educational level, teachers’ instructional language) respectively.

## Results

Table 1 presents descriptive statistics for the key predictors and outcome variables. Children showed enormous variation in their English and Spanish emergent literacy skills (*range* = 32–134 for English and 45–137 for Spanish), with an average standard score of 83.64 (*SD* = 12.84) for English emergent literacy and 94.79 (*SD* = 12.42) for Spanish emergent literacy, suggesting that overall children had better literacy skills in Spanish than in English (*p < *0.001). Notably, the standard scores must be interpreted with caution, because the norm used was based on English-speaking and Spanish-speaking samples.

### Unconditional models

Unconditional models suggested that 73.23% and 82.84% of the variances in English and Spanish emergent literacy skills could be attributed to the differences among individual children, respectively; whereas 26.77% and 17.16% of the variances in English and Spanish emergent literacy skills could be explained by the differences among classrooms, respectively. In the models below, we included a random intercept to account for between-classroom differences.

### Unique contributions of home and classroom language and literacy activities

As shown in Model A (Table 2), neither home nor classroom language and literacy activities uniquely predicted MSHS children’s emergent literacy skills in English, after controlling for demographic covariates and parents’ and teachers’ relative English and Spanish use. However, both the home (*b* = 3.82, *SE* = 1.12, *p* < 0.001, 95% *CI* = [1.63, 6.00]) and classroom (*b* = 3.31, *SE* = 1.43, *p* = 0.021, 95% *CI* = [0.50, 6.12]; see Model C, Table 2) activities uniquely predicted children’s emergent literacy skills in Spanish. Children who experienced more frequent language and literacy activities at home and in the classroom showed better Spanish literacy skills.

**Table 2:** Home and classroom language and literacy activities predicting emergent literacy skills in English and Spanish.

		*Coef.*	*S.E.*	*p*	*95% C.I.*		*Coef.*	*S.E.*	*p*	*95% C.I.*	
English literacy skills (*n* = 232)		Model A					Model B				
	Intercept	**88.76**	**11.83**	**0.000**	**65.57**	**111.95**	26.09	31.25	0.404	−35.15	87.33
*Child level covariates*											
	Child age	−0.06	0.10	0.541	−0.25	0.13	−0.05	0.10	0.603	−0.24	0.14
	Child gender (boy)	**−3.65**	**1.66**	**0.028**	**−6.90**	**−0.40**	**−3.78**	**1.64**	**0.021**	**−7.00**	**−0.56**
	Total # of children at home	−1.22	0.64	0.057	−2.48	0.03	**−1.39**	**0.64**	**0.030**	**−2.64**	**−0.13**
	Parental edu	−0.07	0.22	0.757	−0.49	0.36	−0.10	0.21	0.645	−0.52	0.32
	Parental Spa use	0.10	0.94	0.912	−1.74	1.95	0.16	0.93	0.865	−1.67	1.99
*Classroom level covariates*											
	Teachers’ edu	−0.04	1.04	0.967	−2.08	1.99	−0.07	1.02	0.947	−2.07	1.93
	Teachers’ instructional lang	−0.61	1.03	0.551	−2.63	1.41	−0.64	1.01	0.526	−2.63	1.34
*Key predictors*											
	Home activities	1.40	1.20	0.245	−0.96	3.75	**22.73**	**9.93**	**0.022**	**3.26**	**42.20**
	Classroom activities	0.07	1.73	0.966	−3.31	3.46	**11.70**	**5.64**	**0.038**	**0.65**	**22.75**
*Interaction*											
	Home activities × Classroom activities						**−3.95**	**1.82**	**0.031**	**−7.52**	**−0.37**
											
Spanish literacy skills (*n* = 233)		Model C					Model D				
	Intercept	**60.90**	**10.16**	**0.000**	**40.98**	**80.81**	4.45	28.47	0.876	−51.35	60.25
*Child level control variables*											
	Child age	−0.15	0.09	0.085	−0.32	0.02	−0.14	0.09	0.098	−0.31	0.03
	Child gender (boy)	−1.14	1.53	0.459	−4.14	1.87	−1.25	1.52	0.411	−4.23	1.73
	Total # of children at home	−0.98	0.59	0.097	−2.13	0.18	−1.14	0.59	0.052	−2.30	0.01
	Parental edu	0.10	0.20	0.629	−0.29	0.48	0.07	0.20	0.724	−0.31	0.45
	Parental Spa use	**3.20**	**0.83**	**0.000**	**1.58**	**4.82**	**3.27**	**0.82**	**0.000**	**1.66**	**4.88**
*Classroom level control variables*											
	Teachers’ edu	−0.12	0.77	0.875	−1.64	1.40	−0.13	0.77	0.869	−1.63	1.38
	Teachers’ instructional lang	0.26	0.76	0.733	−1.23	1.76	0.24	0.75	0.747	−1.23	1.72
*Key predictors*											
	Home activities	**3.82**	**1.12**	**0.001**	**1.63**	**6.00**	**22.83**	**9.03**	**0.011**	**5.13**	**40.53**
	Classroom activities	**3.31**	**1.43**	**0.021**	**0.50**	**6.12**	**13.77**	**5.13**	**0.007**	**3.72**	**23.82**
*Interaction*											
	Home activities × Classroom activities						**−3.52**	**1.66**	**0.034**	**−6.77**	**−0.27**

Bolded predictors were significant at the α = 0.05 level.

### Interaction between home and classroom language and literacy activities

In both the English and Spanish models, the interaction term between home and classroom language and literacy activities was significant, indicating that the home and classroom activities moderated the effect of one another on children’s emergent literacy skills (see Models B and D, Table 2).

Specifically, the effect of classroom language and literacy activities was stronger for children who experienced less frequent language and literacy activities at home. For children who engaged in home language and literacy activities almost every day (i.e., 85^th^ percentile or frequency of home activities = 3.6), classroom language and literacy activities did not predict children’s English (*b* = −2.50, *SE* = 2.08, *p* = 0.228, 95% *CI* = [−6.58, 1.57]; upper half of Table 3) or Spanish (*b* = 1.09, *SE* = 1.76, *p* = 0.535, 95% *CI* = [−2.36, 4.55]; lower half of Table 3) literacy skills. However, for children who engaged in home language and literacy activities only 1 to 2 days a week (i.e., 15th percentile or frequency of home activities = 2), classroom activities significantly contributed to children’s Spanish literacy skills (*b* = 6.73, *SE* = 2.15, *p* = 0.002, 95% *CI* = [2.52, 10.93]). Although the conditional effect was non-significant for children’s English literacy skills, the coefficient changed from negative to positive (*b* = 3.81, *SE* = 2.43, *p* = 0.117, 95% *CI* = [−0.95, 8.56]; see Table 3) as the frequency of home literacy activities decreased, revealing a similar trend.

**Table 3:** Conditional effects of home and classroom language and literacy activities on children’s emergent literacy skills in English and Spanish.

		*Coef.*	*S.E.*	*p*	95% *C.I.*	
*Outcome: English literacy skills*						
Effect of classroom language and literacy activities						
	High frequency of home activities (i.e., almost 5–7 days a week)	−2.50	2.08	0.228	−6.58	1.57
	Low frequency of home activities (i.e., 1–2 times a week)	3.81	2.43	0.117	−0.95	8.56
Effect of home language and literacy activities						
	High frequency of classroom activities (i.e., every day)	−0.94	1.61	0.559	−4.09	2.21
	Low frequency of classroom activities (i.e., 3–4 times a week)	2.81	1.36	**0.038**	0.15	5.47
*Outcome: Spanish literacy skills*						
Effect of classroom language and literacy activities						
	High frequency of home activities (i.e., almost 5–7 days a week)	1.09	1.76	0.535	−2.36	4.55
	Low frequency of home activities (i.e., 1–2 times a week)	6.73	2.15	**0.002**	2.52	10.93
Effect of home language and literacy activities						
	High frequency of classroom activities (i.e., every day)	1.71	1.49	0.252	−1.21	4.62
	Low frequency of classroom activities (i.e., 3–4 times a week)	5.05	1.25	**0.000**	2.60	7.50

Bolded effects were significant at the α = 0.05 level.

Similarly, home language and literacy activities had a greater effect on children’s emergent literacy skills, when classroom language and literacy activities were less frequent. For children who engaged in all 8 types of language and literacy activities in the classroom everyday (i.e., 85^th^ percentile or frequency of classroom activities = 6), the effect of home language and literacy activities was non-significant for English (*b* = −0.94, *SE* = 1.61, *p* = 0.559, 95% *CI* = [−4.09, 2.21]) and Spanish (*b* = 1.71, *SE* = 1.49, *p* = 0.252, 95% *CI* = [−1.21, 4.62]; see Table 3) literacy skills. However, for children who engaged in different types of language and literacy activities in the classroom only 3 to 4 times a week (i.e., 15^th^ percentile or frequency of classroom activities = 5.05), home language and literacy activities significantly predicted their English (*b* = 2.81, *SE* = 2.37, *p* = 0.038, 95% *CI* = [0.15, 5.47]) and Spanish (*b* = 5.05, *SE* = 1.25, *p* < 0.001, 95% *CI* = [2.60, 7.50]; see Table 3) literacy skills. Together, these findings suggested that language and literacy activities in the home and classroom settings compensated one another in supporting children’s emergent literacy development.

## Discussion

Children of MSFW families face many challenges in early development and are largely “invisible” in educational research. The current study examined the unique and interactive effects of home and classroom language and literacy activities on MSFW children’s emergent literacy skills. Results showed that the frequencies of home and classroom language and literacy activities uniquely contributed to children’s emergent literacy skills in Spanish, but not in English. Additionally, children’s home and classroom experiences compensated one another. Language and literacy activities in one context were more beneficial for those children who experienced less frequent language and literacy activities in the other context.

### Home language and literacy activities and children’s emergent literacy skills

Consistent with previous work (Farver et al., 2013), the frequency of home language and literacy activities uniquely predicted children’s emergent literacy skills in Spanish, while controlling for demographic variables and children’s classroom experiences. This finding shows the important role of MSFW parents in their children’s emergent literacy development. Even though MSFW parents in our sample had relatively low levels of education (65% of the parents did not have a high school diploma), their engagement in language and literacy activities with the children showed a positive effect on children’s emergent literacy skills in the home language. Indeed, almost 40% of the MSFW parents in our sample reported engaging their children in language and literacy activities 3 to 4 days a week or more frequently, indicating their high motivation and investment in promoting early language and literacy development (Purcell-Gates, 2013).

The frequency of home language and literacy activities did not uniquely predict children’s emergent literacy skills in English. Most MSFW parents reported only or primarily using Spanish with their children. However, this finding does not necessarily mean that the effect of home language and literacy activities is language specific. On one hand, it might take children time to transfer their emergent literacy skills from one language to another, thus showing a delayed cross-language transfer effect. For example, a study found that home literacy activities at kindergarten, which primarily occurred in Spanish, predicted Latino children’s concurrent emergent literacy skills in Spanish, which further predicted their reading skills in both Spanish and English in the 7^th^ grade (Reese et al., 2000). On the other hand, the effect of home language and literacy activities on children’s English emergent literacy skills seems to vary by children’s experiences in the classroom, which will be discussed later.

### Classroom language and literacy activities and children’s emergent literacy skills

Overall, MSHS teachers reported frequently engaging children in language and literacy activities in the classroom. Classroom language and literacy activities uniquely predicted children’s emergent literacy skills in Spanish, highlighting the supportive role of MSHS classroom in children’s home language development. Most MSHS teachers used some Spanish during teaching and learning activities to accommodate the linguistic and cultural needs of MSFW children. Indeed, teachers’ use of the home language of DLLs has been found to benefit children’s home language growth (Collier and Thomas, 2004; Rolstad et al., 2005; Barnett et al., 2007; Figueras-Daniel and Li, 2021).

However, classroom activities did not uniquely predict children’s English emergent literacy skills. This could not be simply explained by teachers’ instructional language(s), as most teachers reported using both English and Spanish during classroom activities. Supplementary analyses examining the interaction between classroom language and literacy activities and teachers’ instructional language(s) suggested that the effect of classroom activities did not vary by the language(s) teachers used (*p*’s > 0.05). One possible explanation is that MSHS teachers might be primarily focusing on supporting children’s English language skills rather than their English literacy skills. Many MSHS children came to the program with very limited English proficiency (Stechuk and Burns, 2005) and might need to acquire adequate English language skills before they could develop emergent literacy skills in English. Additionally, children might be more engaged and interested in Spanish language and literacy activities, which are more relevant to their cultural and linguistic experiences at home and in the MSFW community (Purcell-Gates, 2013). Children’s high level of engagement and interests in learning might further enhance their Spanish learning outcomes (Baroody and Diamond, 2016).

### The compensatory roles of home and classroom language and literacy activities

The effects of home and classroom language and literacy activities compensated one another. Specifically, frequent classroom language and literacy activities predicted children’s Spanish emergent skills for those children who experienced infrequent language and literacy activities at home, but not for those who engaged in home activities 5–7 days a week. These findings were consistent with previous evidence that high-quality early education buffers against the negative effects of impoverished home experiences on children’s language and cognitive outcomes (Magnuson et al., 2004; McCartney et al., 2007; Crosnoe et al., 2010; Vernon-Feagans et al., 2013, 2019). Theoretically, the unique and compensatory roles of home and classroom language and literacy activities support the notion of Mesosystem in the Bioecological Model (Bronfenbrenner and Morris, 2006), which maintains that children’s immediate developmental contexts (e.g., family, school) can interact with one another and jointly impact developmental outcomes.

Likewise, home language and literacy activities benefited children’s emergent literacy skills more when children had less frequent language and literacy activities in the classroom, highlighting the protective role of home language and literacy activities. When children experienced relatively low frequency of classroom activities, home language and literacy activities predicted children’s emergent literacy skills in both Spanish and English. These findings suggested that language and literacy activities in the family context are crucial for MSFW children to develop their home language and may compensate the lack of language and literacy support in the classroom. More interestingly, even though most MSFW parents reported that they predominantly used Spanish with their children, the impact of home language and literacy activities could go beyond the home language to children’s learning of English. Previous work with DLLs also showed that the frequency of family literacy activities in the heritage language predicted children’s vocabulary growth in the societal language when children experienced low to average quality of language stimulation in the classroom (Willard et al., 2021).

### Practical implications

Findings of the study have practical implications for parents and teachers. First, enhancing language and literacy practices in MSFW households may be a valuable strategy to promote children’s development of emergent literacy skills. MSFW parents often view school as the primary context for children to acquire English skills and consider themselves incapable of supporting children’s English learning when cultural and language barriers prevent them from participating in school-related activities (Smith and Johnson, 2019). However, our study showed that home language and literacy activities were associated with children’s emergent literacy skills in *both* English and Spanish, when the frequency of classroom activities was relatively low. Early intervention and prevention programs should encourage MSFW parents to frequently engage in language and literacy practices that are valuable and appropriate in their own cultural and linguistic contexts (Boyce et al., 2010; St. Clair et al., 2012), as well as empower them to recognize their critical role in supporting children’s dual language development.

Additionally, our findings highlight the compensatory role of language and literacy rich MSHS classrooms for children with limited language and literacy resources at home. It is important to promote teachers’ engagement in classroom language and literacy activities via professional training (Solari et al., 2016). Notably, even though classroom is a primary context for English exposure, classroom language and literacy activities did not show a significant effect on children’s English emergent literacy skills. More attention and resources are needed to help MSHS teachers develop effective teaching strategies to support DLLs’ English acquisition (Zepeda et al., 2011). The multisystemic approach recognizes the importance of bridging the gap between the language and literacy practices in the home and school contexts (Barrueco, 2012). Teachers should understand and build upon the literacy knowledge MSFW children gain from their home and community and integrate culturally and linguistically relevant practices into classroom activities (Purcell-Gates, 2013). For instance, in an ethnographic study, compared to book-reading activities, MSFW children were more interested and engaged in culturally responsive activities such as making birthday cards, which they frequently experienced in the migrant farmworker camps (Purcell-Gates, 2013). Interventions should also consider facilitating children’s home and classroom experiences simultaneously (Whitehurst et al., 1994; Grøver et al., 2020). For example, a study in Norway found that providing DLLs with the same set of books to read both in the classroom and at home improved children’s early language and literacy skills in the societal language (Grøver et al., 2020).

### Limitations and future directions

The study has several limitations. First, we only focused on the frequency of language and literacy activities reported by parents and teachers. The adult self-report approach might result in an overestimation of children’s engagement. Observational studies are needed to replicate our findings. Additionally, the study did not examine other types of activities frequently experienced by MSFW, such as reading letters from their extended families, writing a grocery shopping list (Purcell-Gates, 2013), as well as children’s media exposure such as television watching and access to computers and electronic devices (e.g., smartphones). It is also possible that different learning activities contribute to early literacy development in unique ways, an area worth exploring in future research. Furthermore, other important aspects of children’s experiences, such as the quality of language and literacy activities (Justice et al., 2018), the levels of children’s engagement and interest during these activities (Baroody and Diamond, 2016), and the language(s) used during these activities, also play an important role and are worth examining in combination with the frequency of activities. Therefore, more observational work is needed in future research.

In addition to their family members and teachers, MSFW and/or Latino children constantly interact with and gain language and literacy skills from their extended families, peers, and members from the community (Gonzalez and Uhing, 2008; Purcell-Gates, 2013). Future studies should consider these culturally relevant experiences.

Finally, the current study was based on cross-sectional data, which requires us to consider various potential causal scenarios. Children not only learn from language and literacy activities but can also actively elicit language and literacy learning opportunities from their home and classroom environments. Children’s language and literacy experiences might also change over time, as they gain more exposure to the U.S. school system, calling for a longitudinal approach. To date, there is a dearth of longitudinal studies with MSFW children, partially due to the high mobility of the MSFW families (Mathur and Parameswaran, 2012).

## Conclusion

Language and literacy activities in both the home and classroom contexts play an imperative role in MSFW children’s emergent literacy skills. More importantly, frequent language and literacy activities in one context would be more beneficial when children engaged in these activities less frequently in the other context, showing a compensatory relation of the two contexts. Together, these findings can help parents, teachers, and education policy makers find ways to enhance MSFW children’s emergent literacy development, better prepare them for school learning, and ultimately increase equity and equality in early childhood and long-term development.

## Data availability statement

The datasets (Migrant and Seasonal Head Start Study, United States, 2017–2018) for this study can be found here: https://www.icpsr.umich.edu/web/ICPSR/studies/37348. Requests to access these datasets should be directed to the ICPSR online request system.

## Ethics statement

This is a secondary data analysis of an existing dataset. In the original study, written informed consent was obtained from the participating parents, teachers, and children’s legal guardians. The current study was approved by the Institutional Review Board at Rutgers University.

## Author contributions

RL contributed to conceptual framework, data analysis, and manuscript writing. LS contributed to conceptual framework and manuscript editing. All authors contributed to the article and approved the submitted version.

## Conflict of interest

The authors declare that the research was conducted in the absence of any commercial or financial relationships that could be construed as a potential conflict of interest.

## Publisher’s note

All claims expressed in this article are solely those of the authors and do not necessarily represent those of their affiliated organizations, or those of the publisher, the editors and the reviewers. Any product that may be evaluated in this article, or claim that may be made by its manufacturer, is not guaranteed or endorsed by the publisher.
